# Transcriptome analysis reveals key roles of AtLBR-2 in LPS-induced defense responses in plants

**DOI:** 10.1186/s12864-017-4372-4

**Published:** 2017-12-29

**Authors:** Sayaka Iizasa, Ei’ichi Iizasa, Keiichi Watanabe, Yukio Nagano

**Affiliations:** 1 Analytical Research Center for Experimental Sciences, Saga University, Saga, Japan; 2 Department of Biological Resource Sciences, Graduate School of Agriculture, Saga University, Saga, Japan; 3 Department of Biological Science and Technology, The United Graduate School of Agricultural Sciences, Kagoshima University, Kagoshima, Japan; 4 Department of Immunology, Graduate School of Medical and Dental Sciences, Kagoshima University, Kagoshima, Japan

**Keywords:** Plant immunity, Defense response, Lipopolysaccharide, RNA-Seq, *Arabidopsis*, Salicylic acid

## Abstract

**Background:**

Lipopolysaccharide (LPS) from Gram-negative bacteria cause innate immune responses in animals and plants. The molecules involved in LPS signaling in animals are well studied, whereas those in plants are not yet as well documented. Recently, we identified *Arabidopsis* AtLBR-2, which binds to LPS from *Pseudomonas aeruginosa* (pLPS) directly and regulates pLPS-induced defense responses, such as *pathogenesis-related 1* (*PR1*) expression and reactive oxygen species (ROS) production. In this study, we investigated the pLPS-induced transcriptomic changes in wild-type (WT) and the *atlbr-2* mutant *Arabidopsis* plants using RNA-Seq technology.

**Results:**

RNA-Seq data analysis revealed that pLPS treatment significantly altered the expression of 2139 genes, with 605 up-regulated and 1534 down-regulated genes in WT. Gene ontology (GO) analysis on these genes showed that GO terms, “response to bacterium”, “response to salicylic acid (SA) stimulus”, and “response to abscisic acid (ABA) stimulus” were enriched amongst only in up-regulated genes, as compared to the genes that were down-regulated. Comparative analysis of differentially expressed genes between WT and the *atlbr-2* mutant revealed that 65 genes were up-regulated in WT but not in the *atlbr-2* after pLPS treatment. Furthermore, GO analysis on these 65 genes demonstrated their importance for the enrichment of several defense-related GO terms, including “response to bacterium”, “response to SA stimulus”, and “response to ABA stimulus”. We also found reduced levels of pLPS-induced conjugated SA glucoside (SAG) accumulation in *atlbr-2* mutants, and no differences were observed in the gene expression levels in SA-treated WT and the *atlbr-2* mutants*.*

**Conclusion:**

These 65 AtLBR-2-dependent up-regulated genes appear to be important for the enrichment of some defense-related GO terms. Moreover, AtLBR-2 might be a key molecule that is indispensable for the up-regulation of defense-related genes and for SA signaling pathway, which is involved in defense against pathogens containing LPS.

**Electronic supplementary material:**

The online version of this article (10.1186/s12864-017-4372-4) contains supplementary material, which is available to authorized users.

## Background

The endotoxin lipopolysaccharide (LPS), a major component of the outer membranes of Gram-negative bacteria, is one of the most studied pathogen-associated molecular patterns (PAMPs). The perception of LPS triggers various defense responses in plants and animals [1]. In plants, LPS-induced defense responses have been well studied; these include LPS-induced generation of reactive oxygen species (ROS) and nitrogen oxide (NO), salicylic acid (SA) accumulation, expression of *pathogenesis-related* (*PR*) genes, and stomatal closure [2–5]. SA, in particular, is an important signal molecule in plant defense. The accumulation of SA is involved in local defenses as well as in systemic acquired resistance (SAR) [6].

The LPS recognition mechanism has been well studied in animals. In mammals, LPS-binding protein (LBP) and bactericidal/permeability-increasing protein (BPI) play important roles in the regulation of immune responses against LPS [7]. Although both the proteins directly bind to LPS, BPI inhibits whereas LBP enhances the binding of LPS to Toll-like receptor 4, a mammalian LPS receptor. Recently, we identified two *Arabidopsis* LBP/BPI-related proteins, AtLBR-1 and AtLBR-2 [8]. When we incubated recombinant forms of both AtLBR-1 and AtLBR-2 with *Pseudomonas aeruginosa* LPS (pLPS) separately, they exhibited the capability to bind to it directly; *atlbr* mutants showed deficiencies in pLPS-induced *PR1* gene expression and ROS generation. We predicted that AtLBR-2 would be more important than AtLBR-1 in the induction of defense responses to LPS because the binding affinity of AtLBR-2 for LPS appeared higher than that of AtLBR-1, and AtLBR-2 is located in the apoplastic region.

In the present study, we investigated the importance of AtLBR-2 in the dynamic changes in *Arabidopsis* transcriptome in response to LPS treatment. To achieve this goal, we performed a transcriptome analysis using high-throughput mRNA sequencing (RNA-Seq). RNA-Seq analysis using WT and the *atlbr-2-1* identified 65 AtLBR-2-dependent genes that were up-regulated after LPS treatment. These 65 genes appear to be important for the enrichment of some defense-related gene ontology (GO) terms. Our findings highlight the indispensable role of AtLBR-2 in defense signaling mechanism against LPS.

## Results

### Transcriptomic analysis of *P. aeruginosa* LPS-responsive genes in WT *Arabidopsis*

To examine and compare the LPS-induced transcriptional changes between wild-type (WT) and the *atlbr-2-1*, we treated them with LPS from *P. aeruginosa* (pLPS); total RNA was extracted and RNA-Seq analysis was performed.

**Fig. 1:**
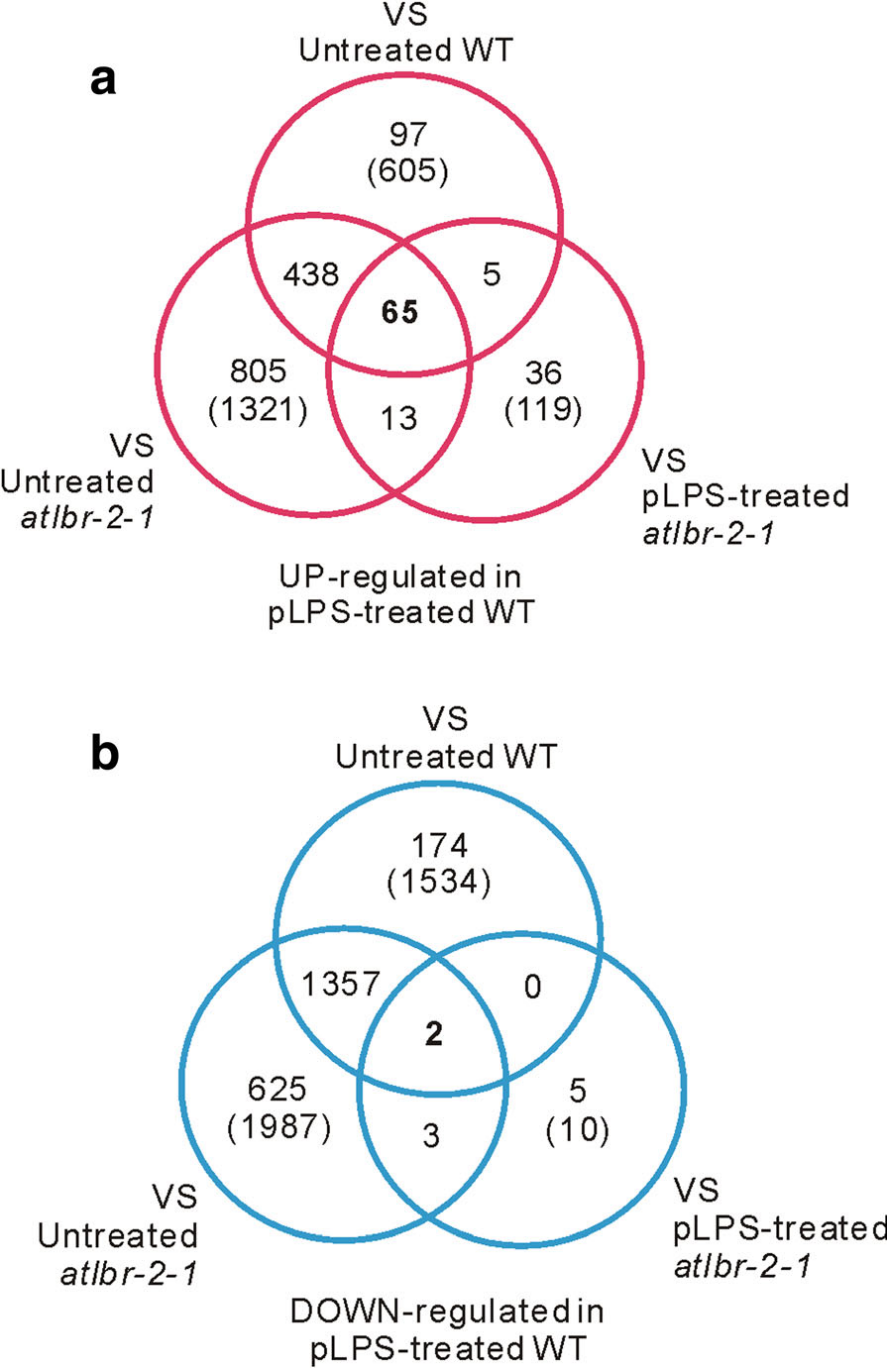
The number of differentially expressed genes in pLPS-treated WT plants. Each RNA-Seq data set obtained from untreated WT, pLPS-treated *atlbr-2-1*, and untreated *atlbr-2-1* plants, were compared with that obtained from pLPS-treated WT plants. The numbers in the parentheses indicate the number of genes identified as up-regulated (**a**) or down-regulated (**b**) in the pLPS-treated WT plants. The numbers of genes, which were up- or down-regulated only in pLPS-treated WT but not in the other three conditions, are indicated in bold type

**Fig. 2:**
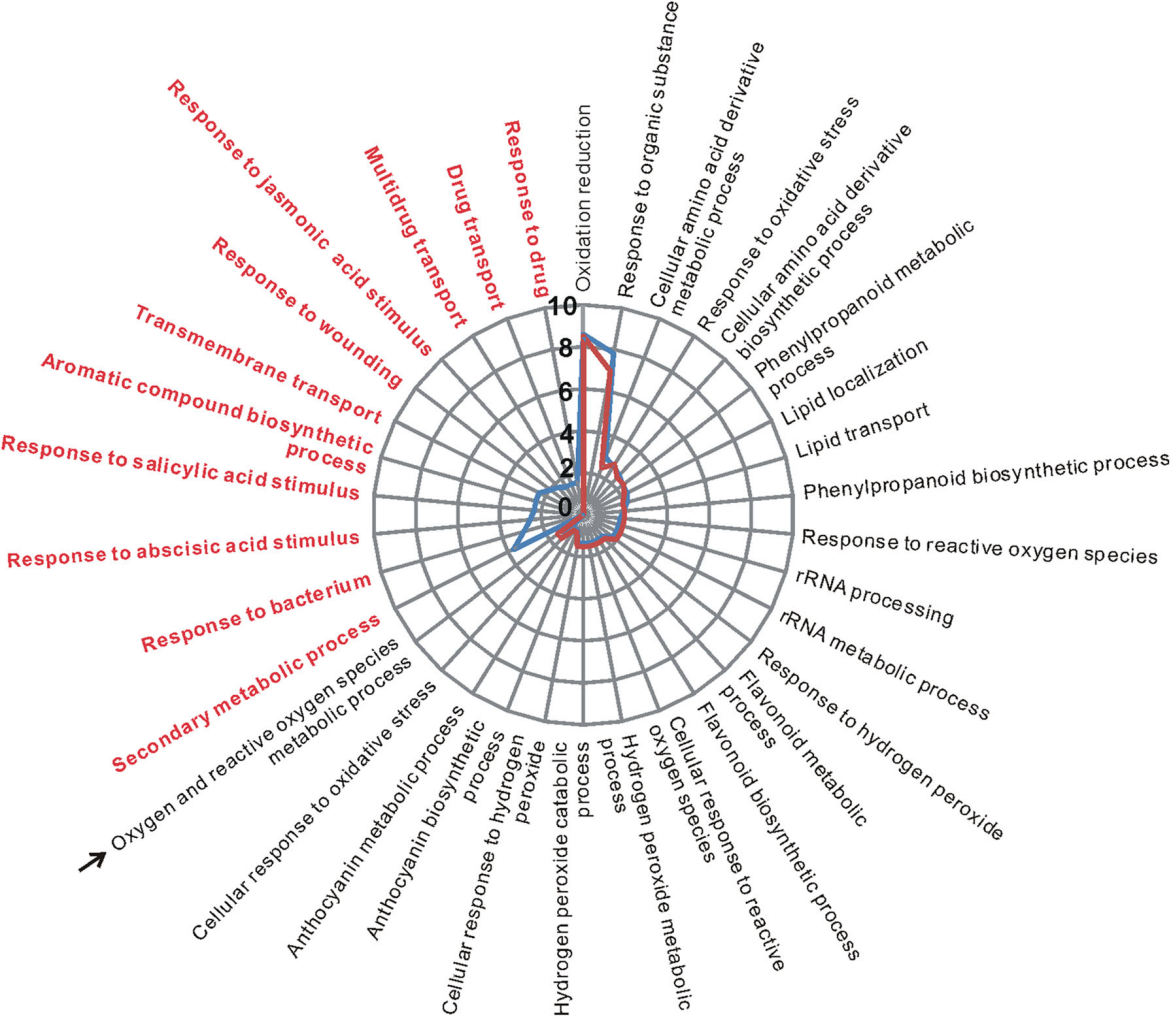
GO classification of pLPS-responsive up-regulated genes. BP GO terms obtained from the GO analysis of 605 pLPS-induced up-regulated genes in WT are shown with the blue line. The same analysis conducted with 540 genes, which excluded 65 AtLBR-2-dependent up-regulated genes from the 605 genes, are shown with the red line. The red font highlights no enriched GO terms in the 540 genes. An arrow indicates the GO term identified only in 540 genes. Scale of y axis shows the percentage of genes that are annotated for each biological process. *P* < 0.01

Firstly, we analyzed the pLPS-responsive genes in the WT. The RNA-Seq data obtained from untreated WT were compared with that of pLPS-treated WT. We observed that the transcript levels of 2139 genes changed significantly in pLPS-treated WT. Of these, 605 genes were identified as up-regulated genes in pLPS-treated WT (Fig. 1a). Moreover, 1534 genes were identified as down-regulated genes in pLPS-treated WT (Fig. 1b). We performed gene ontology (GO) analysis of these genes using the functional annotation chart of DAVID. The biological process (BP) GO classification of the 605 up-regulated genes identified 33 GO terms (*P* < 0.01, Fig. 2, blue line) (Additional file 1: Table S1), including not only defense-related GO terms, but also several metabolic processes-related terms. This finding corresponded with the results reported from transcriptional analysis on *Arabidopsis* seedlings treated with LPS from *Burkholderia cepacia* [9]. Defense-related GO terms included, “response to bacterium”, “response to SA stimulus”, “response to abscisic acid (ABA) stimulus”, “response to jasmonic acid stimulus”, “response to ROS”, and “response to wounding”. In contrast, 1534 down-regulated genes were classified via 43 GO terms (*P* < 0.01) (Additional file 1: Table S2). Interestingly, defense-related GO terms, other than “response to bacterium”, “response to SA stimulus”, “response to ABA stimulus”, were also common in these 43 GO terms. These findings suggested that up-regulation, but not down-regulation, of genes related to bacterial responses may be a characteristic of normal pLPS-induced gene expression. It can also be inferred that SA- and ABA-related pathways may be important for the up-regulation, but not down-regulation, of genes after pLPS treatment.

Furthermore, cellular component (CC) GO analysis showed that 23.0% of the 605 up-regulated genes were categorized as “endomembrane system”; also, 22.9% and 11.9% of the 1534 down-regulated genes were categorized as “endomembrane system” and “intrinsic to membrane”, respectively (Additional file 1: Table S3). These results indicated that genes activated in the membrane-related region were most affected by the pLPS treatment.

### Identification of AtLBR-2-dependent up- or down-regulated genes

To elucidate the importance of AtLBR-2 in pLPS-induced transcriptional responses, we identified the genes that were up-regulated in an AtLBR-2-dependent manner after 24 h of pLPS treatment. We compared each of the three RNA-Seq data with that of pLPS-treated WT (Fig. 1). Furthermore, we studied the genes that were up- or down-regulated only in the pLPS-treated WT plants and not in the other 3 data sets; these were then identified as AtLBR-2-dependent up- or down-regulated genes. A total of 65 candidate genes were identified to be AtLBR-2-dependent up-regulated genes (Fig. 1a, Table 1). We focused on these 65 genes and analyzed them further; only two genes, “*unfertilized embryo sac 11* (*UNE11*: AT4G00080)” and the gene for an “uncharacterized protein (AT3G20340)”, were identified to be AtLBR-2-dependent down-regulated genes (Fig. 1b).

**Table 1:** AtLBR-2-dependent up-regulated 65 genes after pLPS treatment

Accession	Description	Log_2_FC	Ref.
**AT2G14610**	Pathogenesis-related protein 1 (PR1)*	**−5.493297**	[43]
AT3G23120	Receptor like protein 38 (RLP38)*	−4.553003	[44]
AT3G21500	1-deoxy-D-xylulose 5-phosphate synthase 1 (DXPS1)	−4.454822	―
AT2G30770	Putative cytochrome P450 (CYP71A13)*	−3.876201	[17]
AT1G61800	Glucose-6-phosphate/phosphate transporter 2 (GPT2)*	−3.440655	[45, 46]
AT1G21320	Nucleotide binding protein	−3.423526	―
**AT2G14560**	**Late upregulated in response to** ***Hyaloperonospora parasitica*** 1 (LURP1)*	**−3.420434**	[47]
AT4G35180	LYS/HIS transporter 7 (LHT7)*	−3.326263	[44, 46]
AT2G29350	Senescence-associated gene 13 (SAG13)*	−3.152003	[48]
AT4G04510	Cysteine-rich receptor-like kinase (RLK) 38 (CRK38)*	−3.124063	[49]
**AT2G24850**	Tyrosine aminotransferase 3 (TAT3)*	**−2.935669**	[10]
AT2G18660	Plant natriuretic peptide A (PNP-A)*	−2.824188	[50]
AT5G24200	Alpha/beta-Hydrolases superfamily protein*	−2.675765	[51]
AT2G04070	Multidrug and Toxin Extrusion (MATE) efflux family protein	−2.651088	―
AT3G22235	Pathogen and circadian controlled 1 (PCC1)	−2.612637	―
**AT4G12470**	Azelaic acid induced 1 (AZI1)*	**−2.593354**	[52]
AT1G33960	avrRpt2-induced gene 1 (AIG1)*	−2.454032	[53]
AT1G65500	Uncharacterized protein	−2.423526	[45]
AT4G22470	Lipid transfer protein (LTP) family protein*	−2.414268	[54]
AT4G12490	Lipid transfer protein*	−2.37707	[55]
AT4G12480	Early Arabidopsis aluminum induced 1 (pEARLI 1)*	−2.334243	[56]
**AT5G46050**	Peptide transporter 3 (PTR3)*	**−2.322650**	[57]
AT3G28580	P-loop containing nucleoside triphosphate hydrolases superfamily protein	−2.314015	―
**AT3G50480**	Homolog of RPW8 4 (HR4)*	**−2.311148**	[10]
AT2G26400	Acireductone dioxygenase 3 (ARD3)	−2.302582	―
AT1G51820	Leucine-rich repeat protein kinase family protein*	−2.288417	[32]
AT1G02920	Glutathione S-transferase 7 (GSTF7)*	−2.267427	[58]
AT1G65481	Uncharacterized protein	−2.255667	―
AT1G43910	P-loop containing nucleoside triphosphate hydrolases superfamily protein*	−2.252226	[44]
AT4G17660	Protein kinase superfamily protein	−2.126580	―
AT4G26200	1-Amino-cyclopropane-1-carboxylate synthase (ACS7)	−2.091110	―
AT3G63380	Auto-inhibited Ca^2+^-ATPase 12 (ACA12)*	−2.063108	[24, 44]
AT5G09470	Dicarboxylate carriers 3 (DIC3)	−2.058293	―
AT4G12735	Uncharacterized protein	−2.022097	―
AT2G25470	Receptor like protein 21 (RLP21)	−2.002310	―
AT3G26210	Putative cytochrome P450 (CYP71B23)	−1.987360	―
**AT4G23130**	Cysteine-rich RLK 5 (CRK5)*	**−1.985073**	[59]
**AT2G25510**	Uncharacterized protein*	**−1.984502**	[10]
**AT5G03350**	Legume lectin family protein*	**−1.980512**	[10]
AT3G50770	Calmodulin-like 41 (CLM41)*	−1.936221	[55]
**AT4G37990**	Elicitor-activated gene 3–2 (ELI3–2)*	**−1.895395**	[60]
AT4G00170	Plant vesicle-associated membrane protein (VAMP) family protein	−1.809448	―
**AT2G19190**	Flg22-induced RLK 1 (FRK1)*	**−1.804904**	[61]
AT2G20720	Pentatricopeptide repeat (PPR) superfamily protein	−1.790359	―
AT5G44390	FAD-binding Berberine family protein	−1.785875	―
AT1G35230	Arabinogalactan-protein 5 (AGP5)*	−1.779422	[62]
AT5G53870	Early nodulin-like protein 1 (ENODL1)	−1.774478	―
AT2G04050	Multidrug and Toxin Extrusion (MATE) efflux family protein	−1.766112	―
**AT1G02930**	Glutathione S-transferase 6 (GSTF6)*	**−1.750494**	[63]
AT2G43620	Chitinase family protein*	−1.717382	[64]
**AT1G21250**	Cell wall-associated kinase 1 (WAK1)*	**−1.706512**	[10, 65]
AT1G80130	Tetratricopeptide repeat (TPR)-like superfamily protein*	−1.679920	[66]
AT5G44575	Uncharacterized protein	−1.671623	―
AT5G62480	Glutathione S-transferase TAU 9 (GSTU9)	−1.630394	―
AT5G10760	Apoplastic, EDS1-dependent 1 (AED1)*	−1.621488	[50]
AT5G24640	Uncharacterized protein	−1.616614	―
AT5G64000	3′(2′),5′-bisphosphate nucleotidase (SAL2)	−1.600337	―
AT3G28540	P-loop containing nucleoside triphosphate hydrolases superfamily protein	−1.584674	―
AT1G05730	Uncharacterized protein (DUF842)	−1.542038	―
AT1G26420	FAD-binding Berberine family protein	−1.526992	[67]
AT1G67520	Lectin protein kinase family protein	−1.478748	―
AT2G26440	Pectin methylesterase 12 (PME12)*	−1.460767	[68]
AT3G26830	Phytoalexin deficient 3 (PAD3)*	−1.409278	[16, 69]
AT2G41730	Uncharacterized protein	−1.405069	―
**AT1G15520**	Pleiotropic drug resistance 12 (PDR12)*	**−1.361787**	[70]

Genes up-regulated in an AtLBR-2-dependent manner after 24 h pLPS treatment were identified (FDR < 0.01, Log_2_FC < −1.35). Genes, which were related to plant–pathogen interaction or to SA, are indicated by asterisks or in bold type, respectively, with references

### AtLBR-2 is indispensable for pLPS-induced defense-related GO terms

To determine the importance of the 65 AtLBR-2-dependent up-regulated genes in the GO classification of 605 up-regulated genes, we performed GO analysis for 540 genes, excluding the above-mentioned 65 genes from the 605 up-regulated genes (*P* < 0.01, Fig. 2, red line) (Additional file 1: Table S1). Comparing the results of the GO analysis revealed that 540 genes showed no enrichment for defense-related GO terms, including responses to bacterium, SA stimulus, ABA stimulus, wounding, and drug. These results highlight the importance of 65 genes in defense-related GO terms, and demonstrate that AtLBR-2 might be indispensable for the expression of pLPS-induced defense-related genes.

### Characterization of 65 AtLBR-2-dependent up-regulated genes

The details of the 65 pLPS-induced AtLBR-2-dependent up-regulated genes are shown in Table 1. We expected that *PR1* would be one of the 65 AtLBR-2-dependent up-regulated genes, because we previously reported that *atlbr* mutants showed deficiencies in the expression of pLPS-induced *PR1* (AT2G14610) [8]. Consistent with our prediction, as shown in Table 1, *PR1* was the most differentially-expressed gene among the AtLBR-2-dependent up-regulated genes. Furthermore, most of the 65 genes have been reported to be associated with plant–pathogen interaction and with SA-regulated responses.

These 65 genes were also annotated to each of the three GO categories; CC, molecular function (MF), and BP. These genes were assigned the CC GO terms, “endomembrane system” (27.1%), “membrane” (17.1%), and “membrane-bound organelle” (11.4%, Table 2). Thus, approximately 50% of these genes were categorized as membrane-related CC GO terms, highlighting the inter-relationship between the proteins encoded by these genes and AtLBR-2 [8]. Furthermore, the MF GO terms related to catalytic (44.1%), binding (35.3%), and transporter (11.8%) activities were mostly enriched. They were also predicted to participate in 25 BP GO terms, mainly in the “response to stress” (12.0%), “cellular metabolic process” (10.6%), “response to biotic stimulus” (9.2%), and in “response to other organisms” (9.1%). Thus, interestingly, approximately 50% of these genes were involved in response to stress and stimuli. To further define the functions of these 65 genes, the enriched pathways were identified by KOBAS (Table 3). The pathway analysis also revealed that the 65 genes were involved in defense-related pathways, including “camalexin biosynthesis”, “glutathione-mediated detoxification II”, and “plant–pathogen interaction”.

**Table 2:** GO-term-enriched tables of AtLBR-2-dependent up-regulated 65 genes

CC GO term	**%**
Endomembrane system	27.1
Membrane	17.1
Intercellular part	14.3
Membrane-bound organelle	11.4
Intercellular organelle	11.4
External encapsulation structure	8.6
Apoplast	4.3
Membrane part	1.4
Intercellular organelle part	1.4
Organelle membrane	1.4
MF GO term	**%**
Catalytic activety	44.1
Binding	35.3
Transporter activety	11.8
Electron carrier activety	5.9
Structual molecule activety	1.5
BP GO term	**%**
Response to stress*	12.0
Cellular metabolic process	10.6
Response to chemical stimulus*	9.9
Response to biotic stimulus*	9.2
Response to other organism*	9.1
Primary metabolic process	6.3
Transport	5.6
Response to endogeneous stimulus*	4.2
Secondary metabolic process	4.2
Biosynthetic process	4.2
Macromolecule metabolic process	3.5
Immune response*	2.8
Nitrogen compound metabolic process	2.8
Response to abiotic stimulus*	2.1
Regulation of biological process	2.1
Transmembrane transport	2.1
Catabolic process	2.1
Small molecule metabolic process	1.4
Oxidation-reduction process	0.7
Aging	0.7
Establishment of localization in cell	0.7
Multicallular organism reproduction process	0.7
Cell wall organization or biogengesis	0.7
Cellular response to stimulus*	0.7
Cell death	0.7

The 65 AtLBR-2-dependent up-regulated genes were classified by functional categories under the following GO terms: CC (level 2), MF (level 1), and BP (level 2) using the VirtualPlant 1.3 web service. BP GO terms related to stress and stimulus are indicated by asterisks

**Table 3:** Pathway enrichment analysis of 65 AtLBR-2-dependent up-regulated genes

Term	Pathway Database	Database ID	IN	BN	*P*-Value
Camalexin biosynthesis	BioCyc	CAMALEXIN-SYN	3	32	7.44E-05
Glutathione-mediated detoxification II	BioCyc	PWY-6842	3	50	0.000258
Glutathione metabolism	KEGG	ath00480	3	93	0.001468
Cysteine and methionine metabolism	KEGG	ath00270	3	112	0.002458
Plant-pathogen interaction	KEGG	ath04626	3	167	0.007317

A *P*-Value <0.01 was used as a threshold to select significant pathways. *IN* Input number, *BN* Background number

### *atlbr-2-1* mutants showed defect in up-regulation of six pLPS-induced genes

**Fig. 3:**
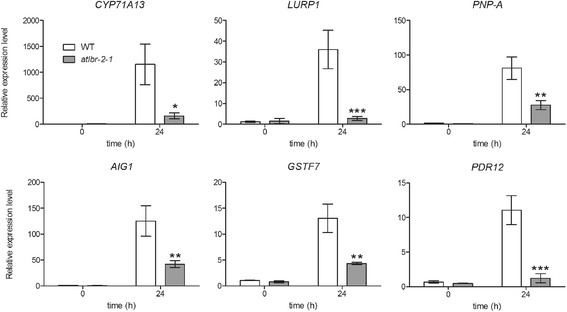
qRT-PCR analysis of six AtLBR-2-dependent up-regulated genes. Among the 65 pLPS-induced AtLBR-2-dependent up-regulated genes, 6 defense-related genes (*CYP71A13*, *LURP1*, *PNP-A*, *AIG1*, *GSTF7*, and *PDR12*) were randomly selected and analyzed by qRT-PCR in WT and *atlbr-2-1* plants treated with pLPS. The mean expression values were calculated from the results of three independent experiments. Means ± standard errors are presented. Significant differences among the means were determined by two-way ANOVA followed by post hoc Bonferroni test compared to WT plants; *P < 0.05, **P < 0.01, ***P < 0.001

To confirm the RNA-Seq results of AtLBR-2-dependent up-regulated genes, we assigned and tested several genes involved in plant–pathogen interaction by conducting quantitative RT-PCR (qRT-PCR**)** using appropriate primers (Additional file 1: Table S4; Fig. 3). In all of the tested 6 genes, putative cytochrome P450 (*CYP71A13*), late upregulated in response to *Hyaloperonospora parasitica* (*LURP1*), plant natriuretic peptide A (*PNP-A*), avrRpt2-induced gene 1 (*AIG1*), glutathione S-transferase 7 (*GSTF7*), and pleiotropic drug resistance 12 (*PDR12*), we could detect the significant differences between WT and the *atlbr-2-1* at 24 h of pLPS treatments, indicating that AtLBR-2-dependent up-regulated genes identified by RNA-Seq were also confirmed by qRT-PCR. However, the expression levels of these 6 genes were not completely abolished in pLPS-treated *atlbr-2-1* mutants. These results suggested the possibility that *AtLBR-1*, the paralog of *AtLBR-2*, may compensate for the absence of the *AtLBR-2* gene. Therefore, we conducted qRT-PCR analysis for these 6 genes with the *atlbr-1* mutant seedlings by the same method that was used on the *atlbr-2-1* mutant (Additional file 1: Figure S1) [8]. Similar to the results shown in Fig. 3, we detected significant differences in all tested genes in pLPS-treated WT and *atlbr-1* mutants, suggesting that AtLBR-1 might also play an important role in some AtLBR-2-dependent up-regulated genes. Furthermore, to exclude the possibility of contamination by other bacterial components, we purified the commercial pLPS. Seedlings were treated with purified pLPS and were analyzed by qRT-PCR (Additional file 1: Figure S2). Similar to the results shown in Fig. 3, we could detect the significant differences between WT and *atlbr-2-1* in all of the genes tested at 24 h of purified pLPS treatments, confirming that this phenomenon was not caused by contamination.

Although we did not observe read counts from the region downstream of the T-DNA insertion site of *AtLBR-2* (Additional file 1: Figure S3), the possibility of slight expression of AtLBR-2 is not excluded because the T-DNA insertion site of the *atlbr-2-1* is located just next to the stop codon of the gene. Thereafter, we conducted qRT-PCR using the cDNA obtained from another pLPS-treated T-DNA insertion line, *atlbr-2-2* [8]. Similar to that in the *atlbr-2-1*, we could detect the significant differences between pLPS-treated WT and the *atlbr-2-2* (Additional file 1: Figure S4).

### Validation of RNA-Seq data by qRT -PCR

**Fig. 4:**
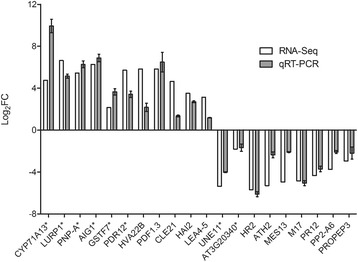
Comparison of RNA-Seq results with those of qRT-PCR. The Log_2_FC values for transcript levels observed in RNA-Seq data (white bar) of randomly selected 20 genes, including AtLBR-2-dependent -independent up- or down-regulated genes, were compared to the results obtained from qRT-PCR (gray bar). AtLBR-2-dependent up- or down-regulated genes are indicated with asterisk: *CYP71A13*, *LURP1*, *PNP-A*, *AIG1*, *GSTF7*, *PDR12*, *UNE11*, and AT3G20340. AtLBR-2-independent up- or down-regulated genes are indicated without asterisk: *HVA22B* (AT5G62490), HVA22 homologue B; *PDF1.3* (AT2G26010), plant defensin 1.3; *CLE21* (AT5G64800), clavata3/ESR-related 21; *HAI2* (AT1G07430), highly ABA-induced 2; *LEA4–5* (AT5G06760), late embryogenesis abundant 4–5; *HR2* (AT3G50460), homolog of RPW8 2; *ATH2* (AT3G47740), *A. thaliana* ABC2 homolog 2; *MES13* (AT1G26360), methyl esterase 13; *M17* (AT2G41260), late embryogenesis abundant gene; *PR12* (AT1G75830); *PP2-A6* (AT5G45080), phloem protein 2-A6; *PROPEP3* (AT5G64905), elicitor peptide 3 precursor

To validate RNA-Seq results, we conducted the expression analysis by qRT-PCR for randomly selected pLPS-responsive genes, including AtLBR-2-dependent or -independent up- or down-regulated genes. Figure 4 shows a comparison between the results from qRT-PCR and RNA-Seq analysis. For all 20 tested genes, transcript levels determined by qRT-PCR analysis were similar to those detected using RNA-Seq, indicating the reliability of the RNA-Seq data.

### A proposed pathway: AtLBR-2-mediated SAG accumulation and following SA-related gene expression

**Fig. 5:**
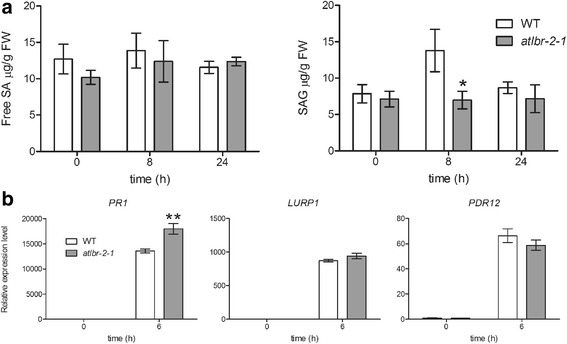
pLPS-induced free SA and SAG accumulation and SA-induced gene expression*.* (**a**) Quantification of the total levels of free SA and SAG were measured by HPLC using samples extracted from *Arabidopsis* treated with pLPS for the indicated time. (**b**) Among the 65 pLPS-induced AtLBR-2-dependent up-regulated genes, the expression of the 3 SA-related genes (*PR1*, *LURP1*, and *PDR12*) were analyzed by qRT-PCR in WT and the *atlbr-2-1* mutants plants treated with SA. The mean values were calculated from the results of three independent experiments. Means ± standard errors are presented. Significant differences among the means were determined by two-way ANOVA followed by post hoc Bonferroni test compared to WT plants; *P < 0.05, **P < 0.01. FW, fresh weight

In the WT, pLPS treatment induced differential expression of many genes related to SA, a potent inducer of pathogen-induced defense responses (Additional file 1: Table S5). Among these, 14 genes were identified as AtLBR-2-dependent up-regulated genes. In addition, 65 AtLBR-2-dependent up-regulated genes were responsible for the enrichment of GO term “response to SA stimulus” (Fig. 2). These RNA-Seq data analyses suggested the association of AtLBR-2 with pLPS-induced SA signaling. Therefore, first, we investigated whether pLPS-induced SA accumulation levels were altered in *atlbr-2-1* plants. Treatment of both WT and the *atlbr-2-1* with pLPS did not result in significant changes in the content of free SA (Fig. 5a, left panel). In contrast, the accumulation levels of conjugated SA glucoside (SAG) between WT and the *atlbr-2-1* showed significant differences at 8 h after pLPS treatment (Fig. 5a, right panel). These results indicated that AtLBR-2 has an important role in pLPS-induced SAG accumulation. Furthermore, to confirm the relationship between AtLBR-2-mediated SAG accumulation and SA-related gene expression, we investigated the expression levels of 3 AtLBR-2-dependent up-regulated genes, *PR1*, *LURP1*, and *PDR12*, in SA-treated WT plants and *atlbr-2-1* mutants by qRT-PCR. These 3 genes are known as SA-related genes, and *atlbr-2-1* mutants showed significant differences in the expression of these genes after pLPS treatment ([8] and Fig. 3). As shown in Fig. 5b, we observed the up-regulation of all tested genes in the SA-treated *atlbr-2-1* to be at the same level, as or more, than that of WT plants. These results suggested that AtLBR-2 may act upstream of the SA signaling (SAG accumulation) pathway induced by pLPS treatment.

## Discussion

Our primary interest in this study was in understanding the role of AtLBR-2 in LPS-induced plant defense responses. In a previous study, we concluded that apoplast-localized AtLBR-2 might play an important role in binding and transferring LPS to the LPS receptor. However, the functional properties of AtLBR-2 have not been characterized in detail. Therefore, in this study, we analyzed, for the first time, the effect of AtLBR-2 on transcriptional changes involved in pLPS treatment using RNA-Seq technology. By our RNA-Seq analysis, we identified that pLPS-induced up-regulated genes were associated with defense-related, as well as metabolism-related processes, which is consistent with a previous study that used LPS from *B. cepacia* [9]. Furthermore, we found a strong association between AtLBR-2 and SA by our RNA-Seq analysis and, in fact, we demonstrated the reduced level of pLPS-induced SAG accumulation in the *atlbr-2-1*, and the SA-induced normal gene expression in *atlbr-2-1* mutants. Our data revealed the importance of AtLBR-2 in SA-mediating signaling pathway in response to LPS or when triggered by LPS.

### AtLBR-2 and SA signaling

In this study, we identified 65 AtLBR-2-dependent genes that were up-regulated after pLPS treatment. The pathway analysis of these 65 genes revealed a significant enrichment of defense-related pathways. In fact, 44 of these genes encode proteins related to defense responses, and 14 genes, including *PR1*, *LURP1*, tyrosine aminotransferase 3 (*TAT3*), azelaic acid induced 1 (*AZI1*), peptide transporter 3 (*PTR3*), homolog of RPW8 4 (*HR4*), cysteine-rich receptor-like kinase (RLK) 5 (*CRK5*), elicitor-activated gene 3–2 (*ELI3–2*), flg22-induced RLK 1 (*FRK1*), glutathione S-transferase 6 (*GSTF6*), cell wall-associated kinase 1 (*WAK1*), *PDR12*, AT2G25510, and AT5G03350, are known to be induced by SA (Additional file 1: Table S5). The SA signaling is mediated by at least two mechanisms, one requiring the NPR1 (NON-EXPRESSOR OF PR1) and the second, which is independent of NPR1 [6]. *PR1* is well known as a marker for NPR1-dependent SA-induced pathway. In addition, Blanco et al. identified some NPR1-dependent SA-induced genes, including *HR4*, *WAK1*, and AT5G03350 by microarray analysis in SA-treated WT and *npr1–1* plants [10]. Furthermore, they also identified NPR1-independent SA-induced genes, including *TAT3* and AT2G25510. These facts, along with our findings in this study, suggested the possibility that AtLBR-2 plays an important role in both NPR1-dependent and **-**independent signaling pathways triggered by LPS. Furthermore, we demonstrated that LPS-induced SAG accumulation was dependent on AtLBR-2. The LPS-induced accumulation of SAG, but not of SA, has been observed in previous studies, and might cause the transient production of SA and the stable accumulation of SAG [11]. In addition, the SA-treated *atlbr-2-1* showed up-regulation of SA-related genes at levels similar to or more than those exhibited by WT plants. These results as well as our previous findings support the hypothesis that AtLBR-2 binds to LPS directly in the apoplastic region of *Arabidopsis* and, subsequently, induces the accumulation of SA (or SAG), leading to the activation of both NPR1-dependent and **-**independent signaling pathways and the gene expression that follows them [8].

Interestingly, *NPR1* expression appeared to be up-regulated after inoculation with *P. syringae* pv. *tomato* (*Pst*) DC3000 or upon SA treatment [12, 13]; however, no difference was observed between the untreated and pLPS-treated WT plants in the present study (FDR < 0.01). The WRKY transcription factors, including WRKY18, WRKY38, and WRKY53, reported to be the targets of NPR1 during SAR [14], appear to be down-regulated in the pLPS-treated WT plants (Additional file 1: Table S5). Shah suggested the existence of a negative feedback loop involving NPR1, which regulates the accumulation of SA [6]. Moreover, in the present study, we showed that SAG concentration returned to the basal levels after 24 h of pLPS treatment. Therefore, based on these observations, we speculated that long-term treatment of pLPS (24 h) might induce SA negative feedback loop involving *NPR1* suppression and result in a decline in SA (SAG) to basal levels.

### AtLBR-2 and camalexin

The 65 AtLBR-2-dependent up-regulated genes included *CYP71A13* and phytoalexin deficient 3 (*PAD3*). These genes encode cytochrome P450 enzymes, which contribute to the enrichment of the “camalexin biosynthesis” pathway. Camalexin is an indole alkaloid phytoalexin produced by *Arabidopsis* that is thought to be important for resistance to necrotrophic fungal pathogens. A previous study revealed the LPS-induced camalexin production in plants [15]. CYP71A13 catalyzes the conversion of indole acetaldoxime to indole-3-acetonitrile, an intermediate in the camalexin biosynthesis [16]. PAD3 catalyzes the conversion of dihydrocamalexic acid to camalexin, which is the last step of camalexin biosynthesis [17]. Interestingly, Nafisi et al. demonstrated that the expression levels of *CYP71A13* and *PAD3* were coregulated in response to infection by *P. syringae* [16]. Furthermore, SA is required for camalexin synthesis, which is mediated by an NPR1-independent pathway [18, 19]. These findings from previous research and the RNA-Seq analysis performed in this study led to the hypothesis that the binding and transfer of LPS to LPS receptor by AtLBR-2 and further SA (SAG) induction might be necessary to activate efficient LPS-induced camalexin biosynthesis. More research is needed to better understand the relationship between AtLBR-2 and camalexin biosynthesis in *Arabidopsis*.

### AtLBR-2 and ATPase

Four genes (AT1G43910, AT3G28540, AT3G28580, and AT3G63380) encoding proteins that function as an ATPase were also among the 65 AtLBR-2-dependent up-regulated genes. Relationship between the four genes and their defense responses have been reported previously. AT1G43910 and AT3G28540 were consistently higher in *sni1*, a transcription repressor of NPR1, when compared with the WT plants [20]. AT3G28580 has been used as singlet oxygen (^1^O_2_)-responsive gene [21, 22]. ^1^O_2_ is a singular ROS that can be produced by phytotoxins during plant–pathogen interactions [23]. In addition, Frei dit Frey showed that AT3G63380 (auto-inhibited Ca^2+^-ATPase 12) cannot interact with FLS2, the bacterial flagellin peptide flg22 receptor, but might contribute to the control of cytosolic Ca^2+^ levels during flg22 responses [24]. Furthermore, a previous report described the relationship between the plasma membrane ATPase of plants and PAMPs-induced rapid extracellular alkalinization [25]. These reports lead us to speculate that AtLBR-2 might be related to LPS-induced ATPase-related responses, e.g. extracellular alkalinization and Ca^2+^ influx/efflux.

### AtLBR-2 and protein kinases

For understanding the LPS recognition mechanism(s), it is essential to investigate the LPS receptor that binds to LPS and initiates a signaling cascade inside the cell via its kinase activity. The well known PAMPs, including flg22, bacterial elongation factor Tu (EF-Tu) peptide elf18, and fungal cell wall component chitin, are recognized by pattern recognition receptors, flagellin-sensitive 2 (FLS2), EF-Tu receptor (EFR), and chitin elicitor receptor kinase 1 **(**CERK1), respectively [26]. Interestingly, the genes induced or repressed by PAMPs are clearly correlated. The flg22 treatment induced the expression not only of EFR and CERK1, in addition to that of FLS2 [27]. In this study, we identified seven AtLBR-2-dependent up-regulated genes, which encode protein kinases (*CRK5*, *CRK38*, *FRK1*, *WAK1*, AT1G51820, AT1G67520, and AT4G17660). The bulb-type lectin S-domain-1 RLK LORE (AT1G61380), which is required for sensing of LPS from *Pseudomonas* and *Xanthomonas* species [28], was not, but lectin protein kinase family protein (AT1G67520) was included in the list. AT1G67520 is known as G-type lectin RLK and is similar to LORE; however, it lacks the transmembrane region [29]. Interestingly, both flg22 and elf26 treatments induced *LORE* and AT1G67520 [27]. These results suggest that AT1G67520 might be involved in recognition of LPS or other related PAMPs. The LPS-responsive S-domain RLK (Nt-Sd-RLK) was also identified in *Nicotiana tabacum* [30]. Signal transduction via RLKs seems to be required for the activation of LPS-induced plant defense responses. We also focused on CRK5, which is potential target gene of WRKY transcription factors. Chen et al. reported that *CRK5* expression was up-regulated by SA treatment and constitutive over-expression of CRK5 led to increased resistance to *Pst* DC3000, which was associated with rapidly induced expression of *PR1* after pathogen infection [31]. Moreover, we also focused on leucine-rich repeat (LRR) protein kinase family protein encoded by AT1G51820, because FLS2, EFR, and their co-receptor brassinosteroid insensitive 1 (BRI1)-associated receptor kinase 1 (BAK1) belong to the LRR-RLK family. Interestingly, AT1G51820 expression was up-regulated after infection with oomycete downy mildew pathogen [32]. These studies suggest that AtLBR-2-related protein kinases might be involved in the perception of PAMP. We speculate on possible LPS perception systems via unknown protein kinases other than LORE or those, which cooperate with LORE.

## Conclusion

In summary, we have reported here the first analysis of the genome-wide effect of AtLBR-2 on pLPS-induced gene expression. The transcriptome analyses performed in this study identify the 65 AtLBR-2-dependent up-regulated genes, and reveal the indispensable role of AtLBR-2 in the up-regulation of pLPS-induced genes associated with defense responses. Further experiments also suggested the existence of an SA-mediated LPS signaling system via AtLBR-2. Thus, we suggest that 65 AtLBR-2-related proteins might be the key candidate molecules in LPS-induced defense mechanisms in plants.

## Methods

### *Arabidopsis* growth conditions and pLPS or SA treatment

*Arabidopsis* ecotype Col-0 was used as the wild-type (WT) plants in this study. All mutants were in the Col background. After 5 d of growth on MS agar plates at 22–24 °C under a 16 h light/8 h dark cycle, the seedlings were transferred to two separate liquid MS medium supplemented with 100 μg/ml LPS from *P. aeruginosa* serotype 10 (pLPS; Sigma-Aldrich Japan, Tokyo, Japan) and 200 μM SA (Wako, Osaka, Japan) that were then kept under continuous light conditions for 24 h and 6 h, respectively. Untreated seedlings were used as controls (0 h). The experiments were performed in three biological replicates.

### RNA sample preparation and high-throughput sequencing

After the treatment, the seedlings were powdered in liquid nitrogen and RNA was extracted using the plant total RNA extraction Miniprep system (Viogene, Taipei, Taiwan) according to the manufacturer’s instructions. The quality of total RNA obtained from pLPS-treated or untreated seedlings was evaluated using the value of RNA Integrity Number (RIN) in the Agilent Bioanalyzer 2100 (Agilent, Santa Clara, CA, USA). The total RNA concentration was measured using a Qubit RNA assay kit (Thermo Fisher Scientific, Waltham, MA, USA). The mRNAs were purified from 100 ng total RNAs by NEBNext Poly(A) mRNA Magnetic Isolation Module (New England Biolabs, Ipswich, MA, USA), according to the manufacturer’s instructions. The sequencing libraries were prepared by NEBNext Ultra Directional RNA Library Prep Kit for Illumina (New England Biolabs, Ipswich, MA, USA), according to the manufacturer’s instructions. The quality of the libraries was assessed by a microchip electrophoresis system (MCE-202 MultiNA; Shimadzu, Kyoto, Japan) and their quantities were measured by Qubit dsDNA BR assay kit (Thermo Fisher Scientific, Waltham, MA, USA). After equimolar amounts of the libraries were pooled, they were used for paired-end read sequencing (2 × 101 bp) on Illumina Hiseq 4000 (Illumina, San Diego, CA, USA).

### Read mapping and transcript assembly

After the quality evaluation and removal of adapter-containing reads, more than 88% left and right reads could be mapped to the *Arabidopsis* TAIR 10 genomes using TopHat software (http://www.ccb.jhu.edu/software/tophat/) (Additional file 1: Table S6) [33]. The concordant pair alignment rate was more than 86%. The transcripts were assembled and fragments per kilobase of transcript per million fragments mapped (FPKM) were estimated using Cufflinks software (http://cole-trapnell-lab.github.io/cufflinks/) (Additional file 2: Table S7) [33].

### Identification of AtLBR-2-dependent up- or down-regulated genes

The differential expression between pLPS-treated WT and the three other data sets (untreated WT, pLPS-treated *atlbr-2-1*, and untreated *atlbr-2-1*) were calculated in R packages, edgeR (https://bioconductor.org/packages/release/bioc/html/edgeR.html) [34] and TCC (http://bioconductor.org/packages/release/bioc/html/TCC.html) [35] using the read counts of each data set, which were calculated by HTSeq-count (https://htseq.readthedocs.io/en/release_0.9.1/index.html) [36]. When compared to RNA-Seq data obtained from pLPS-treated WT, genes with negative Log fold change (Log_2_FC) value changes (FDR; false discovery rate < 0.01, Log_2_FC < −1.35) were identified as up-regulated genes in pLPS-treated WT. In contrast, genes with positive Log_2_FC value changes (FDR < 0.01, Log_2_FC > 1.35) were identified as down-regulated genes in pLPS-treated WT. Commonly up-regulated or down-regulated genes were investigated and 65 and 2 genes were identified as AtLBR-2-dependent up- and down-regulated genes, respectively.

### Gene ontology (GO) enrichment analysis

The GO enrichment analysis for pLPS-induced up- or down-regulated genes in the WT plants was performed using functional annotation charts of DAVID Bioinformatics Resources 6.7 (https://david-d.ncifcrf.gov/) [37]. The distributions of GO terms at levels 1 or 2 for CC, MF, and BP for the 65 AtLBR-2-dependent up-regulated genes were analyzed using the VirtualPlant 1.3 online tool (http://virtualplant.bio.nyu.edu/cgi-bin/vpweb/) [38].

### Pathway analysis

The pathway analysis of the 65 AtLBR-2-dependent up-regulated genes was performed via KOBAS 3.0 (http://kobas.cbi.pku.edu.cn/), which uses the BioCyc (https://biocyc.org/), KEGG (http://www.genome.jp/kegg/), and PANTHER (http://pantherdb.org/) databases [39, 40]. Only pathways with *P*-value <0.01 were listed.

### Real-time PCR (qRT-PCR) analysis

After the pLPS or SA treatment and total RNA isolation, as above, reverse transcription was performed using 1 μg of total RNA [8]. qRT-PCR was run on a PikoReal real-time PCR system (Thermo Fisher Scientific, Waltham, MA, USA), according to the manufacturer’s recommendations using the following conditions: 1 min at 95 °C, and 40 cycles of 5 s at 95 °C and 30 s at 60 °C. Because the FPKM values of *β-tubulin4* (AT5G44340) were not affected by pLPS treatment, it was used as a non-responsive reference gene (Additional file 1: Fig. S5). The sequences of gene-specific primers are mentioned in Table S4 (Additional file 1). Each experiment was repeated at least three times.

### Free SA and SAG measurement

Two-week-old *Arabidopsis* seedlings grown on MS agar plates were transferred to liquid MS medium and treated with 100 μg/ml pLPS for the indicated time points. After the treatment, 80 mg of whole plants were harvested. Both free SA and SAG extraction was performed, based on a previously-reported method [41, 42]. Free SA and SAG were quantified by reverse-phase HPLC on a C18 column (YMC, Kyoto, Japan) and monitored by UV-detection at 240 nm. The column was eluted with 50 to 90% acetonitrile gradient in water containing 0.1% trifluoroacetic acid.

## Additional files

Additional file 1:Table S1-S6. and **Figure S1-S5**. (PDF 579 kb)

Additional file 2: Table S7. The FPKM of all transcripts in each data sets. (XLSX 4687 kb)

## Abbreviations

ABA: abscisic acid
AtLBR-2: *Arabidopsis* LBP/BPI-related 2
BP: biological process
BPI: bactericidal/permeability-increasing protein
CC: cellular component
FDR: false discovery rate
GO: gene ontology
LBP: LPS-binding protein
Log_2_FC: log fold change
LPS: lipopolysaccharide
MF: molecular function
*NPR1*: *non-expressor of PR1*
PAMP: pathogen-associated molecular pattern
pLPS: commercial LPS from *Pseudomonas aeruginosa*
*PR1*: *pathogenesis-related 1*
RNA-Seq: mRNA sequencing
SA: salicylic acid
SAG: conjugated SA glucoside
