# Supplementary material for: Transcriptome analysis reveals key roles of AtLBR-2 in LPS-induced defense responses in plants
Source: BMC Genomics. 2017 Dec 29;18:995. doi: 10.1186/s12864-017-4372-4 (PMC5747113; doi:10.1186/s12864-017-4372-4)

Table S1. Gene ontology (GO) analysis of the 605 and 540 pLPS-induced up-regulated genes from WT plants using the functional annotation chart of DAVID ($P < 0.01$).

BP GO Term	605 genes		540 genes	
	%	***P*-Value**	%	***P*-Value**
Oxidation reduction	8.593750	2.76E-05	8.496732	6.53E-05
Response to organic substance	7.812500	4.32E-04	6.971678	0.007049
Cellular amino acid derivative metabolic process	2.929688	9.50E-04	2.396514	0.002579
Response to oxidative stress	2.734375	0.003404	2.832244	0.003169
Cellular amino acid derivative biosynthetic process	2.539063	3.39E-04	2.396514	0.001554
Phenylpropanoid metabolic process	2.343750	3.33E-04	2.396514	4.65E-04
Lipid localization	2.343750	3.16E-04	2.178649	0.001781
Lipid transport	2.148438	5.02E-04	1.960784	0.003210
Phenylpropanoid biosynthetic process	1.953125	7.52E-04	1.960784	0.001347
Response to reactive oxygen species	1.757813	0.005292	1.960784	0.002375
rRNA processing	1.757813	0.002402	1.960784	0.001042
rRNA metabolic process	1.757813	0.002402	1.960784	0.001042
Response to hydrogen peroxide	1.757813	0.001753	1.960784	7.51E-04
Flavonoid metabolic process	1.367188	7.17E-04	1.525054	3.56E-04
Flavonoid biosynthetic process	1.367188	4.83E-04	1.525054	2.38E-04
Cellular response to reactive oxygen species	1.367188	0.009456	1.525054	0.005058
Hydrogen peroxide metabolic process	1.367188	0.006577	1.525054	0.003471
Hydrogen peroxide catabolic process	1.367188	0.005563	1.525054	0.002919
Cellular response to hydrogen peroxide	1.367188	0.005563	1.525054	0.002919
Anthocyanin biosynthetic process	0.781250	7.38E-04	0.87146	5.01E-04
Anthocyanin metabolic process	0.781250	0.002115	0.87146	0.001447
Cellular response to oxidative stress	1.367188	0.009930	1.525054	0.005322
Oxygen and reactive oxygen species metabolic process	0.000000	—	1.525054	0.009738
Secondary metabolic process	3.710938	0.001235	0.000000	—
Response to bacterium	2.929688	2.42E-04	0.000000	—
Response to abscisic acid stimulus	2.539063	0.008461	0.000000	—
Response to salicylic acid stimulus	2.343750	1.37E-04	0.000000	—

Aromatic compound biosynthetic process	2.343750	0.002747	0.000000	—
Transmembrane transport	2.343750	0.002650	0.000000	—
Response to wounding	1.953125	0.001988	0.000000	—
Response to jasmonic acid stimulus	1.757813	0.009934	0.000000	—
Multidrug transport	1.562500	5.45E-04	0.000000	—
Drug transport	1.562500	8.06E-04	0.000000	—
Response to drug	1.562500	8.68E-04	0.000000	—

Table S2. GO analysis of the 1,534 pLPS-induced down-regulated genes from WT plants using the functional annotation chart of DAVID ($P < 0.01$).

BP GO Term	%	*P*-Value
Response to organic substance	9.309091	1.40E-15
Oxidation reduction	7.781818	3.03E-08
Defense response	7.272727	2.31E-09
Response to endogenous stimulus	7.127273	5.51E-10
Transcription	6.690909	0.003425
Response to hormone stimulus	6.254545	1.23E-07
Intracellular signaling cascade	6.181818	3.73E-10
Response to abiotic stimulus	6.109091	0.005501
Response to oxidative stress	3.490909	2.90E-12
Cellular response to hormone stimulus	3.200000	4.36E-05
Hormone-mediated signaling	3.200000	4.36E-05
Response to carbohydrate stimulus	2.981818	1.47E-13
Immune response	2.909091	8.44E-08
Death	2.690909	5.42E-08
Cell death	2.690909	5.42E-08
Response to auxin stimulus	2.690909	4.25E-05
Innate immune response	2.618182	1.14E-06
Response to chitin	2.472727	9.13E-15
Response to ethylene stimulus	2.181818	8.20E-05
Response to reactive oxygen species	2.181818	1.60E-10
Programmed cell death	2.181818	6.13E-06
Two-component signal transduction system (phosphorelay)	2.109091	1.12E-05
Response to hydrogen peroxide	2.036364	5.08E-11
Ethylene mediated signaling pathway	1.890909	2.97E-06
Apoptosis	1.890909	3.68E-06
Response to water deprivation	1.672727	1.43E-04
Response to water	1.672727	2.94E-04
Cellular response to oxidative stress	1.600000	1.97E-08
Oxygen and reactive oxygen species metabolic process	1.600000	2.01E-07
Cellular response to reactive oxygen species	1.527273	8.45E-08
Cellular response to hydrogen peroxide	1.454545	6.44E-08
Hydrogen peroxide catabolic process	1.454545	6.44E-08

Hydrogen peroxide metabolic process	1.454545	1.16E-07
Response to wounding	1.381818	7.17E-04
Response to heat	1.309091	3.54E-04
Response to jasmonic acid stimulus	1.309091	0.003517
Response to light intensity	1.163636	4.79E-06
Plant-type cell wall organization	1.163636	1.13E-05
Response to high light intensity	0.945455	1.84E-06
Response to glucose stimulus	0.509091	0.001562
Response to hexose stimulus	0.509091	0.001941
Response to monosaccharide stimulus	0.509091	0.001941
Response to fructose stimulus	0.363636	7.76E-04

Table S3. Cellular component (CC) GO analysis of pLPS-induced up- or down-regulated genes from WT plants using the functional annotation chart of DAVID ($P < 0.01$).

**605 pLPS-induced up-regulated genes CC GO Term**	**%**	***P*-Value**
Endomembrane system	23.046875	6.58E-05
External encapsulating structure	4.882813	0.003017
Apoplast	3.320313	0.006991

**1534 pLPS-induced down-regulated genes CC GO Term**	**%**	***P*-Value**
Endomembrane system	22.909091	2.60E-22
Anchored to membrane	2.618182	5.74E-06
Extracellular region	6.472727	1.02E-04
Intrinsic to membrane	11.927273	4.92E-04
Cell wall	3.272727	0.005532
Extracellular space	0.363636	0.006245
Plant-type cell wall	1.745455	0.006290
External encapsulating structure	3.272727	0.007014

Table S4. Primer sets used for quantitative RT-PCR (qRT-PCR).

Set	F/R	Sequence
CYP71A13	F	5′- CCCTCAGTCTCAGGTACGGA -3′
	R	5′- ACCTCTTGAGCTGCTTCACC -3′
LURP1	F	5′- CAGCCCTGTGTGATAGTGGG -3′
	R	5′- CTTACCGTCCGCACTCGTTA -3′
PNP-A	F	5′- TCGGAGGTACAGGGTTCGAT -3′
	R	5′- GCGATAACCCGAAAAGCGTC -3′
AIG1	F	5′- GCTCGCAGCATTGATGAAGG -3′
	R	5′- CTTCTCCTGCGCCTCCATAG -3′
GSTF7	F	5′- CATGTCAGTGCTTGGGTTGC -3′
	R	5′- TAGGGCAATGAGGTCATCGC -3′
PDR12	F	5′- TTCTTTGCCTTGGGTGGTGT -3′
	R	5′- CTCATTGGCTAGGATCGCGT -3′
PR1	F	5′- GGAGCTACGCAGAACAACTAAGA -3′
	R	5′- CCCACGAGGATCATAGTTGCAACTGA -3′
β-Tubulin4	F	5′- GAGGGAGCCATTGACAACATCTT -3′
	R	5′- GCGAACAGTTCACAGCTATGTTCA -3′
HVA22B	F	5′- TGGTCTTACCGGGCATGAAC -3′
	R	5′- TTGCTCCCAAGTCGTCATCC -3′
PDF1.3	F	5′- GCGAGAAGCCAAGTGGTACT -3′
	R	5′- TGTTTTGCCCCCTCAAGGTT -3′
CLE21	F	5′- ACAAGGTTGTGATCACGGAGA -3′
	R	5′- AAGGATTTGGACCTGTGGGG -3′
HAI2	F	5′- GGTGCAAGAGTCTTAGGCGT -3′
	R	5′- CTCAGTCCGATCCGTAACCG -3′
LEA4-5	F	5′- AAGAGAGAAACGCGTCAGCA -3′
	R	5′- GTCCAGTGGTCGAGTGAGTG -3′
UNE11	F	5′- CCGCCACGTCATCAAAAGAC -3′
	R	5′- TGAACGCACGTAACGAGACA -3′
AT3G20340	F	5′- TCAGGCTCACGAAGAAGCAA -3′
	R	5′- ACCCGCGATTGTTCAGGATT -3′
HR2	F	5′- CCACTGATGGCTAAGGTCGA -3′
	R	5′- CGTTTGAGCTCCGCATAAGC -3′

ATH2	F	5′- TCTCCTTCTGCTACCGGTGA -3′
	R	5′- GACCATCTCCTAGGCCATGC -3′
MES13	F	5′- ACCAGCTCGTTGACAAGGAG -3′
	R	5′- GTACCAACACCAAGCTCCGA -3′
M17	F	5′- TGCCACACACGATGAAGTGA -3′
	R	5′- TTGCTGGGGCTCTACAACAG -3′
PR12	F	5′- TGGTGGAAGCACAGAAGTTGT -3′
	R	5′- CACTGATTCTTGCACGCGTT -3′
PP2-A6	F	5′- GGGTGGATATTTTGGCCGGA -3′
	R	5′- TCGTGGGACGTATTGCAACA -3′
PROPEP3	F	5′- CGTCATCACACAGCGAGGAA -3′
	R	5′- TCCTTTTCCTGAACTTGGCGT -3′

Table S5. Up- or down-regulation of SA-related genes in pLPS-treated WT plants. We investigated the differential expression of 44 SA-related genes that had been reported previously. Among them, 25 genes were up-regulated (FDR < 0.01, $Log_2FC > 1.35$) and 19 genes were down-regulated (FDR < 0.01, $Log_2FC < -1.35$) in pLPS-treated WT plants. The genes identified as AtLBR-2-dependent up-regulated genes are indicated by asterisks.

Accession	Gene	Description	Log_2FC	Ref.
AT2G14610	PR1*	Pathogenesis-related protein 1	7.8961	[1]
AT2G14560	LURP1*	Late upregulated in response to *Hyaloperonospora parasitica*	6.6324	[2]
AT5G54610	ANK	Ankyrin-repeat transmembrane protein BDA1	5.7663	[3, 4]
AT1G15520	PDR12*	Pleiotropic drug resistance 12	5.7092	[5]
AT4G37990	ELI3-2*	Elicitor-activated gene 3-2	5.6854	[6]
AT1G12940	NRT2.5	Nitrate transporter 2.5	5.6152	[4]
AT5G46350	WRKY8	WRKY DNA-binding protein 8	4.7893	[7]
AT5G13320	PBS3	4-substituted benzoates-glutamate ligase GH3.12	4.0142	[8]
AT2G19190	FRK1*	Flg22-induced RLK 1	3.8554	[9]
AT1G21250	WAK1*	Wall-associated receptor kinase 1	3.8309	[3, 4]
AT4G14400	ACD6	Accelerated cell death 6	3.7815	[4]
AT2G24850	TAT3*	Tyrosine aminotransferase 3	3.4777	[4]
AT1G48000	MYB112	Putative transcription factor MYB112	3.4516	TAIR
AT4G12470	AZI1*	Azelaic acid induced 1	3.1286	[10]
AT2G25510	AT2G25510*	Uncharacterized protein	3.0497	[4]
AT5G03350	AT5G03350*	Legume lectin family protein	2.7349	[4]
AT4G23130	CRK5*	Cysteine-rich RLK 5	2.6755	[11]
AT3G50480	HR4*	Homolog of RPW8 4	2.2874	[4]
AT1G02930	GSTF6*	Glutathione S-transferase 6	1.8091	[12]
AT4G23210	CRK13	Cysteine-rich RLK 13	1.7417	[13]
AT4G23170	CRK9	Cysteine-rich RLK 9	1.6418	[3]
AT5G54240	CNGC4	Cyclic nucleotide-gated ion channel 4	1.5442	[4]
AT5G46050	PTR3*	Peptide transporter 3	1.5297	[14]
AT1G21270	WAK2	Wall-associated receptor kinase 2	1.5260	TAIR

AT3G09010	AT3G09010	Protein kinase superfamily protein	1.4225	[4]
AT1G17980	PAPS1	Poly(A) polymerase 1	-1.3765	[15]
AT2G47730	GSTF8	Glutathione S-transferase PHI 8	-1.4073	[4]
AT4G23810	WRKY53	WRKY DNA-binding protein 53	-1.4345	[16]
AT3G11340	UGT76B1	UDP-dependent glycosyltransferase 76B1	-1.5205	[4]
AT5G45110	NPR3	NPR1-like protein 3	-1.7294	[17]
AT1G28380	NSL1	Protein necrotic spotted lesions 1	-1.7437	[18]
AT3G25190	AT3G25190	Vacuolar iron transporter homolog 2.1	-1.7665	[4]
AT4G13510	AMT1	Ammonium transporter 1;1	-1.9057	[4]
AT3G01420	DOX1	Alpha-dioxygenase	-1.9216	[3, 4]
AT1G29690	CAD1	Protein constitutively activated cell death 1	-1.9219	[19]
AT5G62470	MYB96	Putative transcription factor MYB96	-1.9701	[20]
AT3G20600	NDR1	Non race-specific disease resistance 1	-2.1589	[21]
AT4G31800	WRKY18	WRKY DNA-binding protein 18	-2.5420	[4, 16, 22]
AT1G80840	WRKY40	WRKY DNA-binding protein 40	-2.9525	[22]
AT3G24500	MBF1C	Multiprotein-bridging factor 1c	-3.3486	[23]
AT2G40000	HSPRO2	HS1 PRO-1 2-like protein	-3.4538	[24]
AT1G07400	HSP17.8-CI	Class I heat shock protein	-3.4960	[4]
AT5G22570	WRKY38	WRKY DNA-binding protein 38	-3.7252	[4, 16]
AT4G25560	MYB18	Putative transcription factor MYB18	-6.6379	TAIR

References

1. Uknes S, Mauch-Mani B, Moyer M, Potter S, Williams S, Dincher S, et al. Acquired resistance in Arabidopsis. Plant Cell. 1992;4:645–56.

2. Loon LC van, Rep M, Pieterse CMJ. Significance of inducible defense-related rroteins in infected plants. Annu Rev Phytopathol. 2006;44:135–62.

3. Blanco F, Garretón V, Frey N, Dominguez C, Pérez-Acle T, Straeten DV der, et al. Identification of NPR1-dependent and independent genes early induced by salicylic acid treatment in Arabidopsis. Plant Mol Biol. 2005;59:927–44.

4. Blanco F, Salinas P, Cecchini NM, Jordana X, Hummelen PV, Alvarez ME, et al. Early genomic responses to salicylic acid in Arabidopsis. Plant Mol Biol. 2009;70:79–102.

5. Campbell EJ, Schenk PM, Kazan K, Penninckx IAMA, Anderson JP, Maclean DJ, et al. Pathogen-responsive expression of a putative ATP-binding cassette transporter gene conferring resistance to the diterpenoid sclareol is regulated by multiple defense signaling pathways in Arabidopsis. Plant Physiol. 2003;133:1272–84.

6. Quirino BF, Normanly J, Amasino RM. Diverse range of gene activity during Arabidopsis thaliana leaf senescence includes pathogen-independent induction of defense-related genes. Plant Mol Biol. 1999;40:267–78.

7. Chen L, Zhang L, Yu D. Wounding-induced WRKY8 is involved in basal defense in Arabidopsis. Mol Plant Microbe Interact. 2010;23:558–65.

8. Nobuta K, Okrent RA, Stoutemyer M, Rodibaugh N, Kempema L, Wildermuth MC, et al. The GH3 acyl adenylase family member PBS3 regulates salicylic acid-dependent defense responses in Arabidopsis. Plant Physiol. 2007;144:1144–56.

9. Yi SY, Shirasu K, Moon JS, Lee S-G, Kwon S-Y. The activated SA and JA signaling pathways have an influence on flg22-triggered oxidative burst and callose deposition. PLOS ONE. 2014;9:e88951.

10. Jung HW, Tschaplinski TJ, Wang L, Glazebrook J, Greenberg JT. Priming in systemic plant immunity. Science. 2009;324:89–91.

11. Chen K, Fan B, Du L, Chen Z. Activation of hypersensitive cell death by pathogen-induced receptor-like protein kinases from Arabidopsis. Plant Mol Biol. 2004;56:271–83.

12. Lieberherr D, Wagner U, Dubuis P-H, Métraux J-P, Mauch F. The rapid induction of glutathione S-transferases AtGSTF2 and AtGSTF6 by avirulent Pseudomonas syringae is the result of combined salicylic acid and ethylene signaling. Plant Cell Physiol. 2003;44:750–7.

13. Acharya BR, Raina S, Maqbool SB, Jagadeeswaran G, Mosher SL, Appel HM, et al. Overexpression of CRK13, an Arabidopsis cysteine-rich receptor-like kinase, results in enhanced resistance to Pseudomonas syringae. Plant J. 2007;50:488–99.

14. Karim S, Holmström K-O, Mandal A, Dahl P, Hohmann S, Brader G, et al. AtPTR3, a wound-induced peptide transporter needed for defence against virulent bacterial pathogens in Arabidopsis. Planta. 2006;225:1431–45.

15. Trost G, Vi SL, Czesnick H, Lange P, Holton N, Giavalisco P, et al. Arabidopsis poly(A) polymerase PAPS1 limits founder-cell recruitment to organ primordia and suppresses the salicylic acid-independent immune response downstream of EDS1/PAD4. Plant J. 2014;77:688–99.

16. Ishihama N, Yoshioka H. Post-translational regulation of WRKY transcription factors in plant immunity. Curr Opin Plant Biol. 2012;15:431–7.

17. Fu ZQ, Yan S, Saleh A, Wang W, Ruble J, Oka N, et al. NPR3 and NPR4 are receptors for the immune signal salicylic acid in plants. Nature. 2012;486:228–32.

18. Noutoshi Y, Kuromori T, Wada T, Hirayama T, Kamiya A, Imura Y, et al. Loss of NECROTIC SPOTTED LESIONS 1 associates with cell death and defense responses in Arabidopsis thaliana. Plant Mol Biol. 2006;62:29–42.

19. Tutsui T, Morita-Yamamuro C, Asada Y, Minami E, Shibuya N, Ikeda A, et al. Salicylic acid and a chitin elicitor both control expression of the CAD1 gene involved in the plant immunity of Arabidopsis. Biosci Biotechnol Biochem. 2006;70:2042–8.

20. Seo PJ, Park C-M. MYB96-mediated abscisic acid signals induce pathogen resistance response by promoting salicylic acid biosynthesis in Arabidopsis. New Phytol. 2010;186:471–83.

21. McDowell JM, Cuzick A, Can C, Beynon J, Dangl JL, Holub EB. Downy mildew (Peronospora parasitica) resistance genes in Arabidopsis vary in functional requirements for NDR1, EDS1, NPR1 and salicylic acid accumulation. Plant J. 2000;22:523–9.

22. Schön M, Töller A, Diezel C, Roth C, Westphal L, Wiermer M, et al. Analyses of wrky18 wrky40 plants reveal critical roles of SA/EDS1 signaling and indole-glucosinolate biosynthesis for golovinomyces orontii resistance and a loss-of resistance towards Pseudomonas syringae pv. tomato AvrRPS4. Mol Plant Microbe Interact. 2013;26:758–67.

23. Suzuki N, Bajad S, Shuman J, Shulaev V, Mittler R. The transcriptional co-activator MBF1c is a key regulator of thermotolerance in Arabidopsis thaliana. J Biol Chem. 2008;283:9269–75.

24. Murray SL, Ingle RA, Petersen LN, Denby KJ. Basal resistance against Pseudomonas syringae in Arabidopsis involves WRKY53 and a protein with homology to a nematode resistance protein. Mol Plant Microbe Interact. 2007;20:1431–8.

Table S6. Mapping results of RNA-Seq reads from pLPS untreated (0 h) and treated (24 h) *Arabidopsis* plants calculated by TopHat software.

Sample		Input	Mapped	Multiple alignments	Mapped percent [%]
WT_0h-1	left		9108355	733630	88.5
	right	10291798	9064807	729268	88.1
	aligned pair		8986163	723249	86.1
WT_0h-2	left		10002374	270397	91.7
	right	10910276	9947049	266488	91.2
	aligned pair		9875713	266488	89.8
WT_0h-3	left		9588682	246155	94.5
	right	10147734	9532742	244181	93.9
	aligned pair		9461137	242512	92.4
WT_24h-1	left		9622012	222224	95.0
	right	10129203	9573317	220570	94.5
	aligned pair		9500722	219105	93.1
WT_24h-2	left		8979667	273784	94.7
	right	9485142	8930638	271879	94.2
	aligned pair		8862851	269952	92.7
WT_24h-3	left		8733723	159468	93.0
	right	9386663	8687862	158361	92.6
	aligned pair		8626426	157141	91.3
*atlbr-2*_0h-1	left		9820040	185958	95.1
	right	10321160	9767160	184238	94.6
	aligned pair		9690135	182947	93.1
*atlbr-2*_0h-2	left		9515306	192782	95.0
	right	10017028	9462903	190974	94.5
	aligned pair		9396537	189689	93.2
*atlbr-2*_0h-3	left		9536469	229329	92.9
	right	10267907	10267907	9474913	92.3
	aligned pair		9474913	227425	91.0
atlbr-2_24h-1	left		10593345	223351	92.8
	right	11418139	11418139	221719	92.3
	aligned pair		10455241	219724	90.7

*atlbr-2*_24h-2	left		11149809	211255	93.3
	right	11953181	11092323	209840	92.8
	aligned pair		11010533	208134	91.5
*atlbr-2*_24h-3	left		10146975	162874	95.4
	right	10639475	10095988	161643	94.9
	aligned pair		10021227	160545	93.5

Figure S1. mRNA levels of 6 AtLBR-2-dependent up-regulated genes in the pLPS-treated *atlbr-1*. The seedlings having a mutation in *AtLBR-1*, a paralog of *AtLBR-2*, were treated with pLPS by the same method described in the Methods section. cDNA obtained from them were analyzed by qRT-PCR. The mean expression values were calculated from the results of three independent experiments. Means ± standard errors are presented. Significant differences among the means were determined by two-way ANOVA followed by *post hoc* Bonferroni test compared to WT plants; *$P < 0.05$, ***$P < 0.001$.

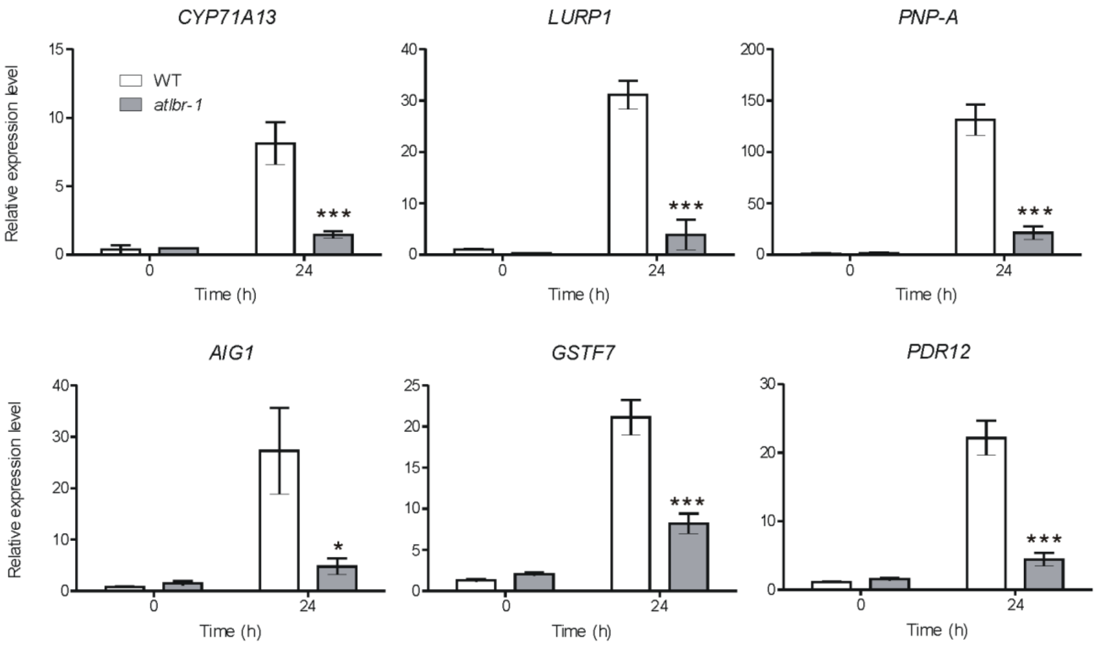

Figure S2. mRNA levels of 6 AtLBR-2-dependent up-regulated genes in seedlings treated with purified pLPS. Purchased pLPS was purified by degradation of nucleic acids and proteins with DNase I, RNase, and proteinase K. mRNA levels of 6 AtLBR-2-dependent up-regulated genes in WT and *atlbr-2-1* seedlings treated with 100 μg/ml purified pLPS during 24 h were detected by qRT-PCR. The mean expression values were calculated from the results of three independent experiments. Means ± standard errors are presented. Significant differences among the means were determined by two-way ANOVA followed by *post hoc* Bonferroni test compared to WT plants; *$P < 0.05$, **$P < 0.01$, ***$P < 0.001$.

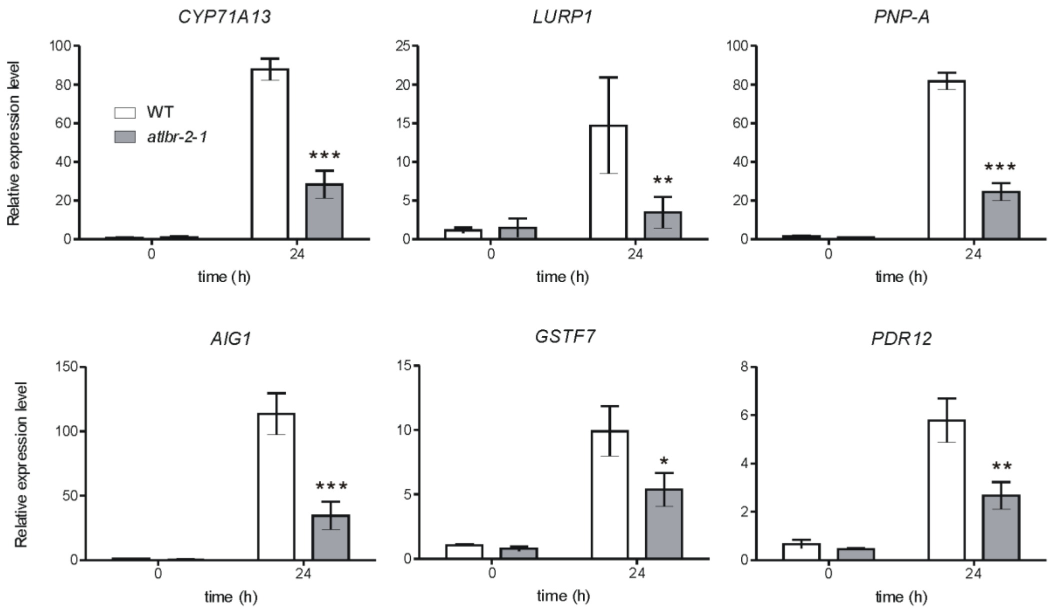

Figure S3. T-DNA insertion effect on the *atlbr-2-1*. Data of RNA-Seq reads from the *atlbr-2-1* T-DNA insertion region were visualized by Integrative Genomics Viewer (IGV).

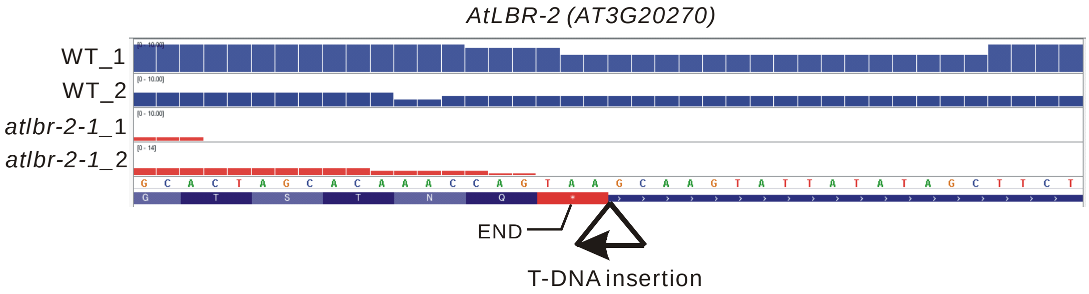

Figure S4. mRNA levels of 6 AtLBR-2-dependent up-regulated genes in the pLPS-treated *atlbr-2-2*. To confirm the RNA-Seq results, cDNA obtained from pLPS-treated or untreated WT and *atlbr-2-2* plants, another T-DNA insertion line of *AtLBR-2*, were analyzed by qRT-PCR. The mean expression values were calculated from the results of three independent experiments. Means ± standard errors are presented. Significant differences among the means were determined by two-way ANOVA followed by *post hoc* Bonferroni test, and compared to WT plants; *$P < 0.05$, **$P < 0.01$, ***$P < 0.001$.

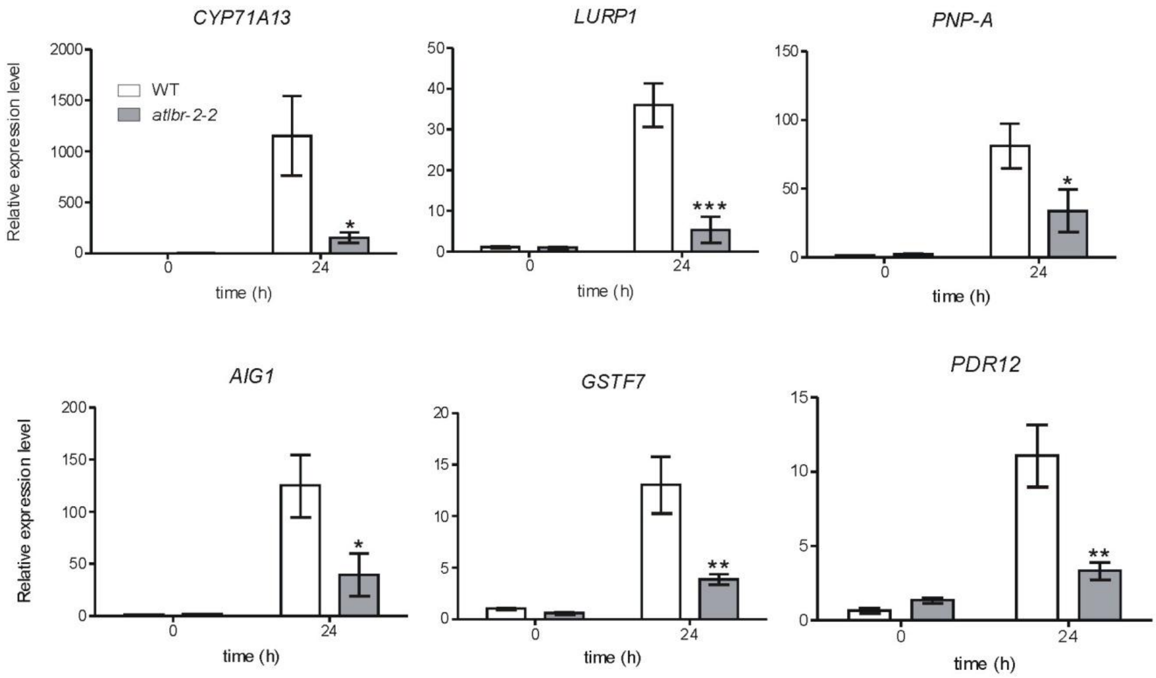

Figure S5. The FPKM values of *β-tubulin4* (AT5G44340) between samples. The FPKM values of *β-tubulin4* were estimated by Cufflinks software. Mean FPKM values were calculated from the results of three biological replicates. Means ± standard errors are presented. Significant differences among means compared each other were evaluated by one-way ANOVA followed by Tukey's multiple comparison test.

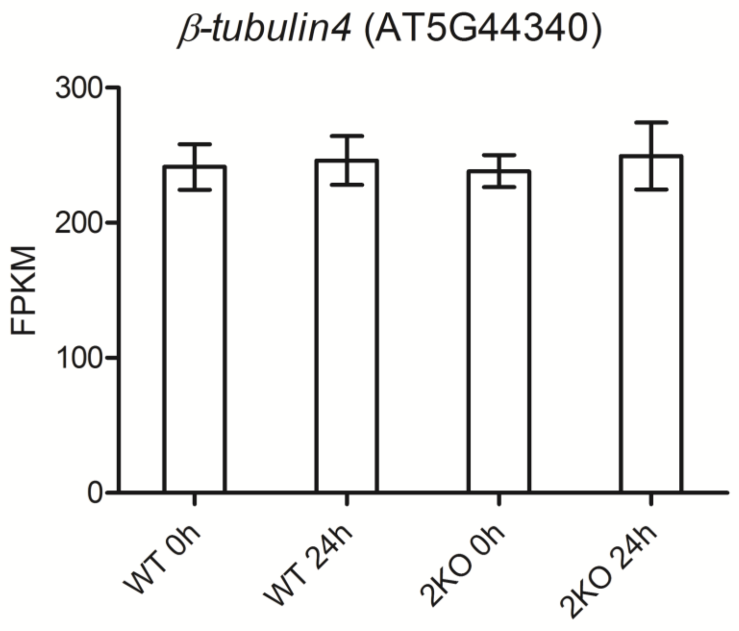

Supplement: Supplementary file 1 — and Figure S1-S5. (PDF 579 kb) [file 12864_2017_4372_MOESM1_ESM.pdf]
